# Implementing new digital options in education/ occupational/ play/ art therapy as tools for self-knowledge, self-managements, self-esteem

**DOI:** 10.1192/j.eurpsy.2021.1808

**Published:** 2021-08-13

**Authors:** C. Emilia

**Affiliations:** Mental Health Center For Children And Adolescents. Stationary Day Neurology And Pediatric Psychiatry., Emergency Clinic Hospital for Children, Cluj-Napoca, România, Cluj-Napoca, Romania

**Keywords:** art therapy, play /occupational therapy, multimedia technology, physical and metaphysical environmen

## Abstract

**Introduction:**

**Figure:**
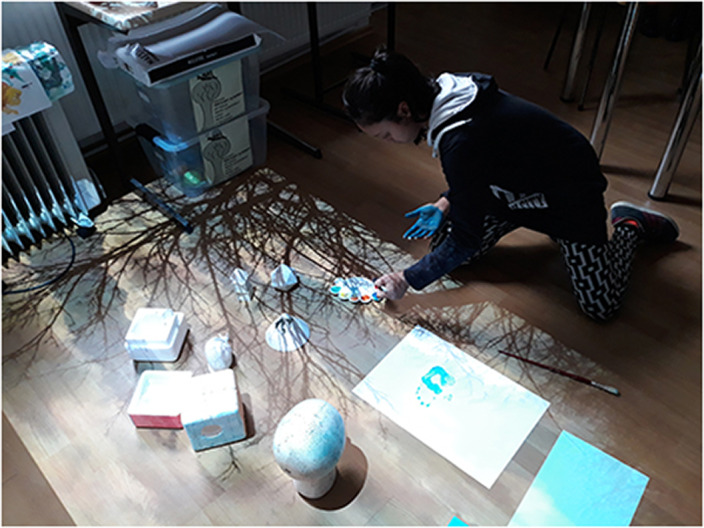

**Figure:**
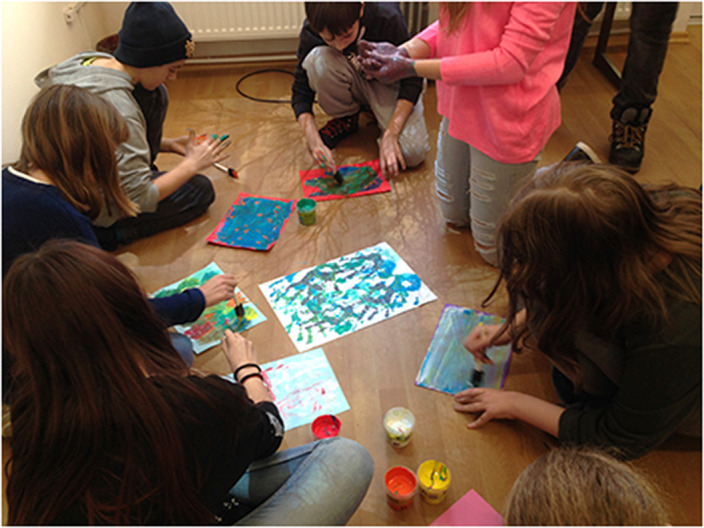

**Figure:**
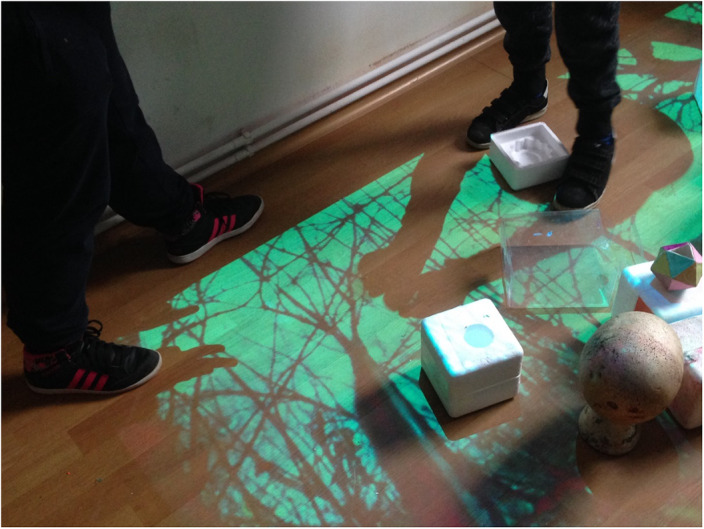

The purpose of art in art-therapy, in this context, is not an exercise of the already acquired knowledge upon the artistic material, but a discovery of the yet unknown. “Multimedia technology, which has evolved into meaningful visual representations, incorporates the science behind human perception and knowledge…Contemporary visual arts bring together, in different degrees of relationship and fusion, fields of art that until now were understood and practiced more individually. The most suitable territory for this partnership is that of the physical and metaphysical environment, provided by the installationist and shareholder arts.” [1].

**Objectives:**

Art-based therapies, as nondirective methods, attempt to visualize past traumatic experiences and harmonize the individual with himself and with others.

**Methods:**

In the preventive activities we include all activities involving nonverbal communication and holistic engagement. ”Beneficiaries can create their own images with which they want to interact, to arrange their environment…We experiment with art-specific ways to make interdisciplinary exchanges and cultural interferences using the universal language of visual arts along with intercultural elements and religious ecumenism … Sometimes, common themes with schools and higher education are addressed as an extra-curricular complement. Benefits are multiple, diverse and complex, appear on the paths that offer inter/pluri/transcultural learning opportunities and exchange of knowledge, making space and time connections between different cultures. [2]

**Results:**

The creative process and the interaction increases self esteem, courage, taking of risks, the learning of new skills.

**Conclusions:**

New ideas, conceptions and ways of expression emerge, enriching the patient’s life according with the therapeutic purposes. [3].

**Disclosure:**

REFERENCES [1] Chirila, Emilia (2011), PhD Thesis, Educaţie artistică şi art-terapie cu mijloace specifice ceramicii [Artistic Education and Art-therapy within the Specific Means of Ceramics], University of Art and Design, Cluj-Napoca, Romania, p. 390

